# Effect of the muscle nanostructure changes during post-mortem aging on tenderness of different beef breeds

**DOI:** 10.5713/ajas.20.0488

**Published:** 2020-10-14

**Authors:** Zimkhitha Soji

**Affiliations:** 1 Department of Livestock and Pasture Science, University of Fort Hare, Alice 5700, South Africa

**Keywords:** Cooking Loss, Muscle Fibre Diameter, Muscle Fibre Spacing, Myofibril Diameter, Myofibril Spacing, Sarcomere Length

## Abstract

**Objective:**

Tenderness is a very complex feature, and the process of its formation is very complicated and not fully understood. Its diversification is one of the most important problems of beef production, as a result beef aging is widely used to improve tenderness as it is believed to provide a homogeneous product to consumers. While few studies have evaluated the muscle structure properties in relation to tenderness from early post-mortem, there little to no information available on how the muscle nanostructure of beef carcasses changes during post-mortem ageing to determine the appropriate aging time for acceptable tenderness.

**Methods:**

Muscle nanostructure (myofibril diameter [MYD], myofibril spacing [MYS], muscle fibre diameter [MFD], muscle fibre spacing [MFS], and sarcomere length [SL]), meat tenderness and cooking loss [CL]) were measured on 20 A2 *longissimus* muscles of Bonsmara, Beefmaster, Hereford, and Simbra at 45_mins_, 1, 3, and 7 days post-slaughter. Muscle nanostructure was measured using a scanning electron microscope, while tenderness was measured using Warner Bratzler shear force.

**Results:**

At 45 minutes post-slaughter, breed affected MYD and MYS only, while at 24_hrs_ it also affected MFD and MFS. On day 3 breed effected MFS and SL, while on day 7 breed effected tenderness only. As the muscles matured, both MYD and MYS decreased while CL increased, and the muscles became tender. There was no uniformity on muscle texture features (surface structure, fibre separation, muscle contraction, and relaxation) throughout the ageing period.

**Conclusion:**

Meat tenderness can be directly linked to breed related myofibril structure changes during aging in particular the MYD, spacing between myofibrils and their interaction; while the MFD, spacing between muscle fibres, SL, and CL explain the non-uniformity in beef tenderness.

## INTRODUCTION

In several international consumer surveys it has been highlighted that regardless of animal species, the most significant meat quality attributes include tenderness, juiciness and flavour [1–3] with tenderness being the most appreciated attribute by consumers particularly in beef [4–8]. In view of this, producers recommended that accurate prediction of meat tenderness is very important for supplying consistent eating quality of meat products as this results in market premiums [9]. Nonetheless, tenderness is the most difficult attribute to predict. Meat tenderness is highly variable and is affected by the meat cut and a range of intrinsic and extrinsic factors associated with the conversion of muscle to meat [7,10,11]. Swatland [12] also highlighted that it becomes a challenge to improve tenderness through genetics if the nanostructure components such as sarcomere length (SL), muscle fibre orientation and fibre texture are not known.

In attempts, therefore, to predict meat tenderness through muscle nanostructure, Zhao et al [13] indicated that beef tenderness is a complex trait that can further be dictated by three structural elements of the muscle which include muscle fibres, connective tissue and intramuscular fat present in the muscle. Soji and Chulayo [14] further indicated that muscle tenderness is enhanced by muscle fibre bundle characteristics at approximately 24 hours post-slaughter; with the myofibrillar structure at 45 mins post-slaughter being a good predictor of the required aging period for individual breeds to further enhance tenderness.

Post-mortem aging is a value adding-process that has been expansively practised globally by the meat industry for years [15]. Beef aging in particular is widely used to improve tenderness and to provide a homogeneous product for consumers [16–20]. Various aging processes are practised from traditional carcass hanging to vacuum packing of portion-cuts for a certain duration of cooling storage [15]. Of these, vacuum packing at refrigerated temperatures is the most commonly used aging system by the meat industry and has been reported to provide retailers with a way to increase tenderness [21–23]. Nonetheless there is little information available on how the muscle nanostructure of beef carcasses changes during post-mortem ageing. As a result, Pulford et al [24,25] have long been trying to determine the appropriate aging time to obtain acceptable tenderness. Thus the diversification of tenderness is still one of the most important problems of beef production as tenderness is a very complex feature and the process of its formation is not fully understood. The aim of this study, therefore, was to evaluate how the muscle nanostructure components (myofibril diameter [MYD], myofibril spacing [MYS], muscle fibre diameter [MFD], muscle fibre spacing [MFS], and SL) of beef carcasses from different breeds affect and/or enhance meat tenderness during post-mortem aging of (0, 1, 3, 7 days).

## MATERIALS AND METHODS

### Ethical clearance

Consent to carry out the study was granted by the University of Fort Hare Research Ethics Committee (UFH/UREC) with reference number: MUC411SSOJ01.

### Study site description

The study was conducted at a high throughput abattoir in East London at the Buffalo City Metropolitan Municipality of the Eastern Cape Province, Republic of South Africa (RSA). The abattoir operates under the standard commercial abattoir procedures and slaughters up to 1,000 livestock units per day. It complies with the stipulations of the Meat Safety Act (Act No.40 of 2000) [26] which created an official system of meat inspection to provide measures in promoting meat safety and the safety of animal products; As well as regulations set by the Agricultural Products Standards Act (Act No 119 of 1990) for classification of meat intended for sale in the republic of South Africa [27].

### Study animals, handling and slaughter procedure

Twenty (n = 20) A-class steers from four different breed types (Bonsmara, 5; Beef master, 5; Hereford, 5; and Simbra, 5) were used in the study. The studied animals were transported from different South African feedlots and were all from a radius of within 120 km (travelling distance). On arrival at the abattoir, they were kept in lairages overnight to ease travelling stress. Handling of animals from the farm of origin to the slaughterhouse complied with all relevant national standards and legislation, whereby; it was ensured that loading and off-loading was carried-out in accordance with the Code for Transport and Handling of Livestock. The code specifies that livestock should be loaded and off-loaded as quickly as possible and with minimal physical handling, prodding or undue stress. Lairage Facilities shall be suitable for their purpose and their size and the construction shall be adequate for the number of livestock therein and holding pens shall not be overcrowded, i.e. livestock shall be able to lie down. Appropriate protection from adverse weather conditions such as excessive heat, sun, wind and cold shall be provided. Fresh and clean water should also be provided *ad libitum* to the animals. All these protocols were followed from farm and at the abattoir.

The animals were then all humanely slaughtered on the following day following the commercial standard procedures. For the purpose of this study, all animals used were of the same age (A class), fat class (fat class 2), conformation (medium score 3), sex (steers) and had no traces of bruises. These classification characteristics were selected since they are considered as ideal classes in the South African meat market. The classification was in accordance with Act no 119 of 1990 (Agricultural Product Standards Act).

### Meat sample harvesting

After humane slaughter carcasses were dressed and split into half at the slaughter line and immediately stored in cold rooms (±4°C) at approximately 45 minutes post-slaughter. A portable digital fibre-optic pH meter (Model HQ11d United States of America) was used to measure pH directly on the carcasses at 45 minutes, 24 hours, 3 and 7 days post-slaughter. Simultaneously a small incision was also made on the carcasses to harvest an approximately 20 g subsection of the *longissimus thoracis et lumborum* (LTL) muscle on the left side of each carcass between the 10th rib and the third lumbar vertebra. The LTL muscle was immediately immersed into a 3% formalin for muscle nanostructure analysis, while 100 mm thick samples were also collected following the same procedure but vacuum packed into impermeable plastic bags for tenderness and cooking loss (CL) measurements. At approximately 24 hours post-slaughter other smaller subsections of the LTL muscle (20 g) were harvested and immediately immersed into 3% formalin for further muscle nanostructure analysis, while other 100 mm thick samples were also harvested for tenderness and CL measurements. The same process continued at 3 and 7 days.

### Determination of muscle nanostructure

The 20 g LTL samples which were harvested for the muscle nanostructure were immediately put in small bottles containing 3% formalin for fixation. They were then transported to the Botany laboratory of the University of Fort Hare for approximately 2 hours in cooler box filled with ice. During the analysis, the samples were dehydrated to remove formalin and kept in ethanol for 20 minutes in an ascending order of 10% up to 100% respectively. In order to improve electrical conductivity of the sample surface in the scanning electron microscope (SEM), a thin film of gold palladium was used for sputter coating to enhance the analysis.

Critical point drying was performed using the Hitachi critical point dryer HCP-2 (Hitachi Koki Co Ltd, Tokyo, Japan) to prevent the samples from alteration and to boost good structural preservation. This was done by mounting the samples on aluminium stubs with double-sided carbon tape then the sputter coating with gold-palladium (Au-Pb) using the Eiko IB.3 Ion Coater (EIKO Engineering Co TD, Tokyo, Japan). The samples were then observed under the JEOL JSM-6390LV SEM for the determination of the skeletal surface area of beef muscles. The nanostructure of the skeletal surface area for the samples was then viewed using JEOL JM-5600 SEM at ×5,000 magnification on 0, 1, 3, and 7 days aged meat samples.

### Meat tenderness and cooking loss measurements

Warner Bratzler shear force (WBSF) measurements were done on 100 mm steaks that were stored and/or frozen for 45 minutes, 24 hours, 3 and 7 days at −20°C refrigerator temperature. After each freezing and/or storage period, samples were left to thaw at 25°C, room temperature (measured using an analogue thermometer) for approximately 10 hours. The samples were weighed before cooking and thereafter re-labelled and placed in water-tight PVC plastic bags for cooking in a water bath (Model TRH) for 45 minutes to a final internal temperature of 71°C. After cooking the samples were cooled down at room temperature for approximately ±20 minutes and then reweighed. The CL was measured using a formula by Honikel [28] as follows:

$$Cooking\;loss\;(CL)\;\%=\left[\frac{weight\;before\;cooking-weight\;after\;cooking}{weight\;before\;cooking}\right]\times 100\%$$

Following cooking, from each sample three sub samples of approximately 12.7 mm core diameter were cored parallel to the grain of the meat. The samples were sheared perpendicular to the fibre direction using a Warner Bratzler shear device mounted on an Instron 3344 Universal Testing Apparatus. Cross head speed at 400 mm/min, one shear in the centre of each core. WBSF was measured as the peak force (Newtons) average for three cores per sample.

### Statistical analysis

Data was analyzed using general linear model procedure (PROC GLM) of JMP 9.0 [29] to test the effect of aging on the muscle nanostructure characteristics (MYD, MYS, MFD, MFS, and SL), CL and tenderness of different breeds. Significant means (p<0.05) were separated using the least significant difference method. The following model was used:

$${\text{Y}}_{\text{ijk}}=\mu +\alpha_{\text{i}}+{ɛ}_{\text{ijk}}$$

Where; Y _ijk_ = response variable (MYD, MYS, MFD, MFS, SL, CL, and tenderness); μ = overall mean; α_i_ = effect of aging period; ɛ_ijk_ = random error.

The strength of relationships between muscle nanostruc ture properties, CL, and tenderness were tested using Pearson’s correlation coefficient of XLSTAT version 2018.5 statistical software for excel.

## RESULTS AND DISCUSSION

When comparing the studied breeds in each day it was observed that at 45 minutes post-slaughter, there were breed related changes on the myofibril structure (Table 1); where Bonsmara had the largest MYD and differed significantly from all other breeds. Also while the MYD of Simbra was similar to that of Hereford and Beef master, Hereford and Beef master differed from each other with Beef master recording the smallest MYD. Furthermore, Simbra and Bonsmara had similar and largest MYS respectively, and they differed from Beef master and Hereford which recorded the lowest and similar MYS.

At 24 hours post-slaughter, breed related changes in the muscle fibre bundle characteristics were observed in addition to the myofibril structure changes. While all other breeds were similar, Beef master differed and recorded the smallest MYD. Similar results were also observed on the MYS of Beef master. Beef master also recorded the smallest MFD and was only similar to Simbra while Simbra was similar to Bonsmara and Hereford with Bonsmara recording the largest MFD. Furthermore Bonsmara and Simbra had a similar MFS, likewise Hereford and Beef master were similar, with beef master recording the largest MFS and Bonsmara recording the smallest.

On day 3, breed related changes were evident on MFS and SL only. Beef master and Hereford were similar and recorded the largest MFS while Bonsmara and Simbra were also similar and recorded the smallest MFS. On the other hand Beef master, Bonsmara and Simbra recorded the longest SL and were similar while Hereford recorded the shortest SL. On day 7, breed related changes were only evident on WBSF values where differences were only observed between Beef master and Hereford while all other breeds were similar. Beef master recorded higher WBSF while Hereford recorded the lowest WBSF values.

The present results indicate that at early post-mortem the myofibril structure of each breed changes, while at 24 hours in addition to the myofibril structure changes, the muscle fibre bundle characteristics also change and differ in each breed. Furthermore on day 3 in addition to the muscle fibre bundle characteristics changes in each breed, the SLs among breeds also change, while on day 7 breed related changes are observed only on WBSF values. These results are in agreement with Khasrad et al [30] who indicated that breed influences the muscle structure. The breed related changes on the myofibril structure early post-mortem can be attributed to post-mortem proteolytic activity which enhances the breakdown of the myofibril structure protein [31–33] and breeds become susceptible to differently. These proteins maintain the structural integrity of the myofibrils and once degraded, the rigid structure of the myofibrils deteriorates [34]. Although according to Huff-Lonergan et al [32]; Veiseth-Kent et al [35] this is usually expected during post-mortem storage in the course of the tenderisation phase, in the present study the degradation of the myofibril structure occurred immediately after slaughter but this degradation was not directly linked to tenderness at early post-mortem.

Furthermore, after 24 hours post-slaughter, while the myo fibril structure degradation was stabilising, some muscle fibre bundle characteristics started to deviate in the present study. In particular the MYD, MYS and MFD stabilised on day 3 while variations in the MFS still progressed among breeds. Hughes [36] and Pulford et al [24] indicated that the extent of muscle structural changes post-mortem depends on the rate and extent of pH decline. The pH of the muscle can affect the MYD, MYS, MFD, and the distance between the actin and myosin filaments within the myofilament lattice [36]. The pH of the studied muscles was more or less the same across all breeds in each day, pH related changes were only evident on the myofibril structure at 24 hours post-slaughter in the present study and there was no uniformity on how they changed. These myofibril structural changes can be related to susceptibility of different breeds to the extent of pH decline early post-mortem, this further explains why the myofibril structure began to stabilise on day 3.

Also on day 3 variations in SL among different breeds were evident. These results contradict those reported by Modika et al [37] suggesting that meat tenderisation begins immediately after slaughter. The present results suggest that immediately after slaughter, there is a breed related enzymatic proteolytic system activation which can be associated with the muscle nanostructure degradation but not directly linked to tenderness since differences in tenderness related attributes such as SL were evident from day 3. This can further be justified by the changes in the muscle nanostructure properties which become completely constant while WBSF differed among breeds only at day 7 in the present study. Although the WBSF values in the present study were relatively low, indicating that the tenderness of all breeds was acceptable, the results substantiate that at early post-mortem breed influences the muscle nanostructure changes, but the changes in the muscle nanostructure early post-mortem may not be directly linked to tenderness, although they may provide a good prediction of which breed is likely to be tender faster than the others during post-mortem storage, agreeing with the results of Soji and Chulayo [14].

When looking across the aging period for each breed, the MYD of Beef master was constant up to day 3 and started to deviate on day 7 where it decreased more significantly than the other days. Bonsmara MYD decreased throughout the aging period. Hereford MYD decreased between days 0 and day 1 while it was constant from day 3 to day 7. Also the MYD of Simbra was constant between day 0 and 1, decreased significantly between day 1 and 3, but was also constant between day 3 and 7. Notably Bonsmara MYD differed from those of the other breeds throughout the aging period, while similarities among other breeds were evident across some days.

Moreover, the MYS of Beef master, Bonsmara and Here ford was constant between days 0 and 1, decreased significantly between day 1 and 3, but was constant between days 3 and 7. Simbra MYS differed between days 0 and 1, but was constant between days 3 and 7. Notably Simbra MYS differed from those of other breeds throughout the aging period, while similarities among other breeds were evident across some days.

In terms of the muscle fibre bundle characteristics, the MFD of Hereford and Simbra did not change throughout the aging period. However, notable differences were observed between day 1 and 7 for Beef master and Bonsmara where Beef master MFD increased throughout aging while Bonsmara MFD decreased. On the other hand the MFS of Beef master decreased significantly between day 0 and day 1, but was similar from day 1 to 7. Notable MFS differences were observed between day 1 and day 3 for Bonsmara where the MFS decreased significantly although there was no uniformity across other days. Hereford had a constant MFS across all days, likewise the MFS of Simbra was similar throughout the aging period.

Differences on SL of Beef master during the ageing period were between day 0 and 1 as well as day 1 and 7, with the SL decreasing between day 0 and 1 but increased from day 3 onwards. The SL differences were observed between day 1 and 7 only for Bonsmara with the SL decreasing throughout the aging period. Although the SL of Hereford was more or less similar throughout the aging period, it decreased from day 0–3 and drastically increased from day 7. For Simbra, SL differences were observed between day 0 and 1 as well as between day 0 and 3 with the SL decreasing from day 0–1 and increased from day 3–7.

The CL for Bonsmara was similar from day 0–1 as well as from day 3–7 while these groups differed significantly from each other. The CL of Bonsmara on day 0 differed from all others while similarities were observed between day 1 and 3 as well as between day 3 and 7. A similar trend was observed for Hereford. For Simbra, similarities were only observed between days 3–7. The WBSF values of beef master were similar between days 0–1 as well as between days 3–7, while on Bonsmara similarities were only seen between days 3 and 7. Hereford and Simbra on the other hand had similar WBSF between day 0–1 and WBSF differed from day 1–7.

Notably the CL and myofibril characteristics (MYS and MYD) decreased with aging while tenderness increased across all breeds. On the other hand there was no uniformity on the changes of muscle fibre bundle characteristics (MFD and MFS) and SL during aging. Furthermore interaction between aging and breed were significant only for MYD (p = 0.0003), MYS (p = 0.0129), and MFS (p = 0.0087).

The results of the present study suggest that the aging effect on the muscle nanostructure, tenderness and CL is dependent on breed. The results agree to those of Cifuni et al [38] who also indicated that aging is dependent on various factors which include breed, metabolic status and environmental factors like the rearing system and stressors. Notably aging had a strong influence on the MYD, MYS, SL, CL, and tenderness. As the muscles matured both MYD and MYS decreased while CL increased and tenderness improved with the extent of change depending on each breed. These results agree with Prates et al [39] who indicated that the weakening of the myofibril structure, with the consequent transversal fragmentation of sarcomeres is a major structural change in myofibrils occurring during meat aging. This justifies why the myofibril structure changes (MYD and MYS) early post-mortem could not be directly linked to tenderness in the present study but were identified as good predictors of aging period through the rate of myofibril degradation, to improve tenderness for individual breeds.

Table 2 further shows that aging had a strong negative re lationship with MYD, MYS, and tenderness while it had a strong positive relationship with CL. This means that as aging period increases, the myofibril structure weakens (decrease in MYS and MYD) resulting in improved tenderness (decrease in WBSF values), while the CL increases. This can be attributed to water translocation from within the myofibril space to intermyofibril space and finally to inter and extrafascia spaces [40]. This movement of water results in the formation of gaps between muscle fibres, fibre bundles and the primysial network influencing drip and purge losses [40].

Subsequently, during post-mortem aging the loss of water from the muscle, or changes in the localised distribution of water, may affect the rate of heat transfer and the hardening or softening of protein structures during cooking, resulting ultimately in changes to the textural properties of meat during cooking [41] hence there was high CL in the present study. Therefore, there are important changes in the distribution of water within the muscle that can have a significant effect in the ultimate meat texture, and these changes should be considered during meat aging. This partially explains why aging can improve tenderness but cannot resolve the issue of non-uniformity in tenderness.

The WBSF on the other hand also had a strong positive relationship with MYD and MYS with the WBSF also decreasing as the MYD and MYD decrease during aging resulting in improved tenderness. These results agree with Wyrwisz et al [42] who indicated that the changes in the structural integrity of myofibrils during maturation result in improved meat tenderness.

**Figure 1:**
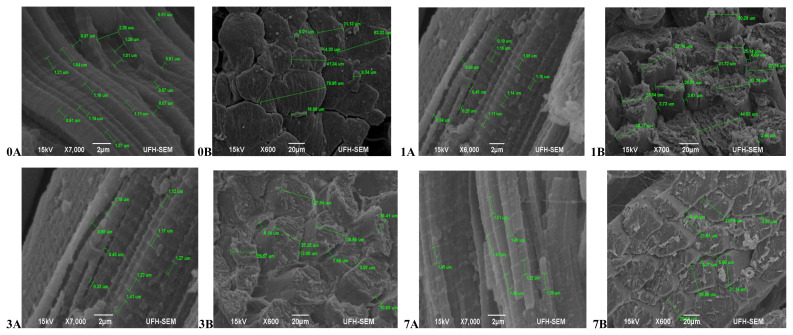
Transverse and Lateral muscle nanostructure changes of Bonsmara during a 7 day ageing period; observed under the JEOL JSM-6390LV scanning electron microscope (SEM) and further viewed using JEOL JM-5600 SEM at ×5,000 magnification. A, Lateral muscle structure from day 0, 1, 3, and 7 ageing period; B, transverse muscle structure from day 0, 1, 3, and 7 ageing period. μm, micrometre.

**Figure 2:**
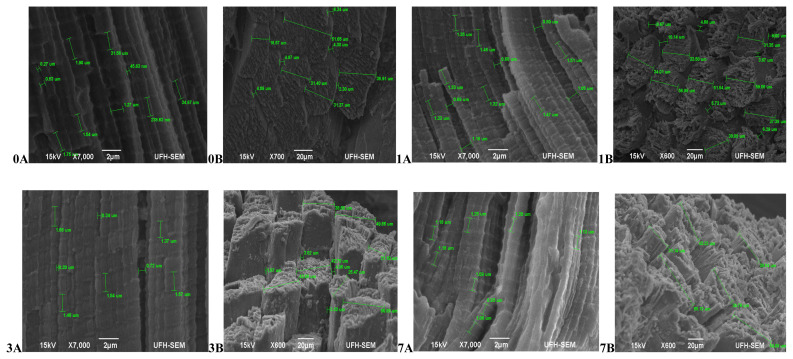
Transverse and Lateral muscle nanostructure changes of Beef master during a 7 day ageing period; observed under the JEOL JSM-6390LV scanning electron microscope (SEM) and further viewed using JEOL JM-5600 SEM at ×5,000 magnification. A, Lateral muscle structure from day 0, 1, 3, and 7 ageing period; B, transverse muscle structure from day 0, 1, 3, and 7 ageing period. μm, micrometre.

In terms of the muscle texture features (Figure 1), at 45 mins post-slaughter (day 0) Bonsmara had relaxed and elongated muscles (0A) with a smooth surface structure and slight separation between muscle fibre bundles (0B), while at 24 hours (day 1) the muscles contracted (1A) with muscle fibres visibly separated and a coarse surface structure (1B). On day 3, the muscles remained contracted (3A) with the surface structure also coarse but separation between fibre bundles was more visible (3B). On day 7, the muscles fibres were more defined, relaxed (7A) and slightly coarse (7B). Beefmaster (Figure 2) at 45 mins post-slaughter (day 0) had less defined muscle structure with a slightly coarse surface structure. The surface structure became extremely coarse during aging with muscle fibres starting to pull off on the on day 7 while the muscle length was non-uniform throughout the ageing period.

**Figure 3:**
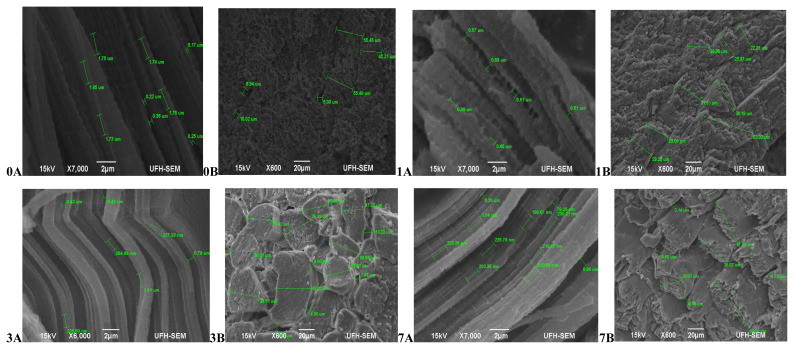
Transverse and Lateral muscle nanostructure changes of Hereford during a 7 day ageing period; observed under the JEOL JSM-6390LV scanning electron microscope (SEM) and further viewed using JEOL JM-5600 SEM at ×5,000 magnification. A, Lateral muscle structure from day 0, 1, 3, and 7 ageing period; B, transverse muscle structure from day 0, 1, 3, and 7 ageing period. μm, micrometre.

**Figure 4:**
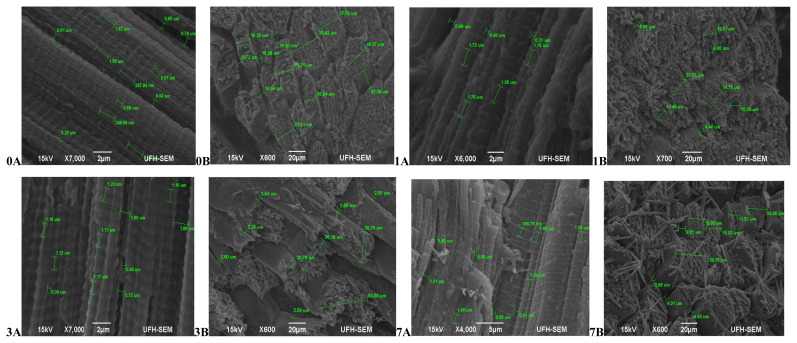
Transverse and Lateral muscle nanostructure changes of Simbra during a 7 day ageing period; observed under the JEOL JSM-6390LV scanning electron microscope (SEM) and further viewed using JEOL JM-5600 SEM at ×5,000 magnification. A, Lateral muscle structure from day 0, 1, 3, and 7 ageing period; B, transverse muscle structure from day 0, 1, 3, and 7 ageing period. μm, micrometre.

Hereford (Figure 3) had more relaxed muscle fibres at 45 mins post-slaughter (day 0A) with a coarse surface structure and no separation between muscle fibre bundles (0B). At 24 hours (day 1) muscles were contracted (1A) and still with no separation between muscle fibres (1B). The muscles became more contracted on day 3 (3A) with a smooth surface structure and slight separation between muscle fibre bundles (3B). Muscles began to relax again on day 7 (7A), however the surface structure became coarser with abundant separation between muscles fibre bundles and fibres starting to pull off (7B).The muscle structure of Simbra (Figure 4) on the other hand became coarser during aging with fibre bundles becoming thicker and fibres pulling off till the 7th day. There was no uniformity in the muscle relaxation during the ageing period but more relaxed muscles were observed on day 1 and 7 only.

The results from the present study indicate that through out the aging period, the pattern of muscle nanostructure differs among different breeds, agreeing to Listrat et al [43] who also highlighted that breed influences the muscle structure. However, there was no uniformity on how the muscle texture features which include the surface structure, fibre separation, and the mechanism of muscle contraction and relaxation changed during the aging period in the present study. These muscle texture features are directly linked to the MFD, MFS, and SL and although the MFD, MFS, and SL were less effective in tenderness improvement during aging in the present study, the lack of uniformity on these muscle nanostructure features during aging could explain why ageing improves meat tenderness but cannot resolve the non-uniformity in beef tenderness.

Therefore, in as much as the meat grading and classifica tion systems have been questioned in terms of their eligibility to resolve the issue of tenderness, differences in tenderness within the same grade/class animals can always be detected due to the lack of uniformity on how the muscle nanostructure of each breed changes during aging.

## CONCLUSION

The present study suggests that for up to 7 days of aging period the complexity in beef tenderness can be directly linked to breed related myofibril structure changes in particular the myofibril diameter, spacing between myofibrils and their interaction; while the muscle fibre diameter, spacing between muscle fibres and SL could explain the non-uniformity in beef tenderness. Further research should put emphasis on finding the exact aging period beyond 7 days that could stabilise the non-uniformity in beef tenderness and provide acceptable tenderness to consumers. However, the meat industry needs to also consider that aging is a long process when compared to the rate at which meat should be supplied to consumers and breed is a very complex factor especially when interrelated with tenderness. Thus alternatively, the non-uniformity in beef tenderness could be resolved through different innovative cooking methods and ingredients such as marinates that could be suggested to consumers.

## Figures and Tables

**Table 1:** Muscle nanostructure, tenderness and cooking loss of South African A2 beef breeds during 7 days of ageing at −20°C

Day	Breed	MYD	MYS	MFD	MFS	SL	CL	WBSF
0	Beef master	1.27^aD^	1.11^aBC^	4.32^aAB^	2.88^aBC^	1.63^aBC^	3.70^aAB^	3.93^aGH^
	Bonsmara	1.58^cG^	1.23^bC^	4.43^aABC^	3.09^aBC^	1.65^aC^	3.69^aAB^	4.00^aH^
	Hereford	1.47^bF^	1.17^aC^	4.42^aABC^	2.76^aAB^	1.56^aABC^	3.65^aA^	3.92^aGH^
	Simbra	1.38^abEF^	1.33^cD^	4.37^aAB^	2.90^aBC^	1.65^aC^	3.59^aA^	3.96^aGH^
1	Beef master	1.22^aD^	1.02^aB^	4.21^aA^	2.39^aA^	1.46^aA^	3.87^aBCD^	3.88^aFG^
	Bonsmara	1.39^bEF^	1.18^bC^	4.78^bCDE^	3.36^bC^	1.54^aABC^	3.98^aDEF^	3.80^aFG^
	Hereford	1.34^bE^	1.14^bC^	4.65^bBCD^	2.75^aAB^	1.49^aA^	3.89^aD^	3.82^aFG^
	Simbra	1.38^bEF^	1.15^bC^	4.46^abABC^	2.83^aABC^	1.47^aA^	3.96^aDE^	3.74^aDEFG^
3	Beef master	1.21^aDC^	0.88^aA^	4.52^aABC^	3.31^bC^	1.57^bABC^	4.08^aEFGHI^	3.54^aCD^
	Bonsmara	1.14^aBC^	0.88^aA^	4.45^aABC^	2.67^aAB^	1.54^bABC^	4.16^aFGHI^	3.34^aBC^
	Hereford	1.15^aBC^	0.88^aA^	4.41^aABC^	3.11^bBC^	1.43^aA^	4.04^aDEFG^	3.54^aDC^
	Simbra	1.18^aDBC^	0.88^aA^	4.32^aAB^	2.81^aAB^	1.52^abAB^	4.08^aEFGHI^	3.48^aC^
7	Beef master	1.05^aAB^	0.88^aA^	4.72^aCDE^	2.96^aBC^	1.60^aBC^	4.22^aHI^	3.39^bBC^
	Bonsmara	1.03^aA^	0.88^aA^	4.33^aAB^	2.82^aAB^	1.52^aAB^	4.26^aH^	3.19^abAB^
	Hereford	1.10^aAB^	0.88^aA^	4.45^aABC^	2.89^aBC^	1.55^aABC^	4.21^aHI^	3.09^aA^
	Simbra	1.09^aAB^	0.88^aA^	4.62^aBCD^	3.03^aBC^	1.55^aABC^	4.17^aHI^	3.18^abAB^
SE		0.038	1.023	4.212	0.177	0.043	0.067	3.879

MYD, myofibril diameter; MYS, myofibril spacing; MFD, muscle fibre diameter; MFS, muscle fibre spacing; SL, sarcomere length; CL, cooking loss; WBSF, Warner Bratzler shear force (newtons); SE, standard error.

a–c Means in the same day among different breeds with different letters are significantly different (p<0.05).

A–I Means across different aging periods among breeds are significantly different (p<0.05).

**Table 2:** Correlations among nanostructure, tenderness and cooking loss

Variables	Ageing	MYD	MYS	MFD	MFS	SL	CL	WBSF
Ageing	**1**	**−0.769**	**−0.740**	0.115	0.050	−0.062	**0.747**	**−0.829**
MYD		**1**	**0.733**	0.026	0.070	0.081	**−0.631**	**0.713**
MYS			**1**	−0.016	0.060	0.192	**−0.684**	**0.701**
MFD				**1**	**0.258**	0.162	0.061	−0.053
MFS					**1**	0.212	−0.019	−0.065
SL						**1**	−0.135	0.047
CL							**1**	**−0.662**
WBSF								**1**

Values in bold are different from 0 with a significance level α = 0.05.

MYD, myofibril diameter; MYS, myofibril spacing; MFD, muscle fibre diameter; MFS, muscle fibre spacing; SL, sarcomere length; CL, cooking loss; WBSF, Warner Bratzler shear force.
